# Is obesity a risk factor for skeletal muscle ageing?

**DOI:** 10.18632/aging.101941

**Published:** 2019-04-29

**Authors:** Cameron Hill, Jason Tallis

**Affiliations:** 1 Randall Centre for Cell and Molecular Biophysics, School of Life Sciences, London, SE1 1UL, United Kingdom; 2 Conventry University Centre for Sport, Kings College London, Coventry, West Midlands SE1 1UL, United Kingdom

**Keywords:** isolated muscles, obesity, sarcopenic obesity, power, force, muscle quality

The proportion of the European population aged >65 years is expected to be 25% by 2030, with 20% of the global population projected to be obese by 2030 [1]. Understating the interaction between ageing and obesity has become an important area of research given the increased risk of serious health conditions and diseases. Given the importance of skeletal muscle in metabolism, completing tasks of daily living and being physically active, it is of importance to better understand the concomitant effects of obesity and old age on skeletal muscle health and performance. Evidence has demonstrated that ageing and obesity in younger adults independently alter the contractile performance of skeletal muscle, resulting in impaired locomotor and respiratory muscle function. Both ageing and obesity independently have been associated with chronic inflammation, muscle atrophy and reduced myogenesis, fibre type shifting and impaired excitation-contraction coupling [1,2], providing a strong mechanistic justification to support an obesity-induced exacerbation of the typical muscle ageing process. Consequently, sarcopenic obesity, characterised by a high fat mass and low muscle mass, has been demonstrated to contribute to a greater risk of developing type 2 diabetes, cardiovascular diseases, and has been linked to reduced longevity of life [2]. Despite this, the body of evidence directly examining the synergism between obesity and ageing on muscle function is lacking and controversial.

The effects of ageing on the contractile function of skeletal muscle has been thoroughly investigated, categorised by a reduction in both isometric strength and concentric power when quantified in both absolute and relative terms [3]. The effect of obesity on contractile function is a growing area of interest, with results being far less conclusive compared to the ageing literature. A recent review has demonstrated that obesity effects on contractile performance are muscle specific, with evidence for a reduction in both force and power normalised to body mass, and a reduction in muscle quality (performance relative to muscle size) [4]. Contrary to the typical ageing response, there is evidence indicating that obesity may increase the absolute force producing capacity of the postural muscles; a response to an elevated overload stimulus due to an increased body mass. Given that the magnitude of this response is lower than the increase in body weight, this positive adaptation would be insufficient in offsetting the decline in whole body functional performance.

Based on this understanding, if obesity effects on skeletal muscle function were to complement the responses seen with ageing, an additive effect would result in more substantial implications for locomotor function and health-related fitness. The small body of evidence that has explored this indicates poorer stair ascent and descent, impaired balance, a slower more tentative gait pattern and difficulties moving (activities of daily living) in sarcopenic obese individuals compared to aged lean counterparts [5]. Moreover, studies of muscle function in old obese adults report elevated absolute strength of antigravity muscles, but equivocal evidence regarding muscle quality [6]. Although it would seem intuitive to assume that an elevated body mass and aged, weakened musculature would account for the outlined biomechanical changes, the effect of obesity directly at the muscle is currently unclear.

Studies examining the contractile performance of isolated muscle have been important in informing our understanding of the muscle ageing response and have more recently been used to provide insight into the effect of obesity on muscle function. An isolated muscle model allows researchers to quantify direct muscle responses, distinguish fibre type-specific differences and allow a more accurate measurement of muscle quality. It has also been proposed that isolated muscle models provide a more accurate measure of fatigue resistance when comparing obese and normal weight experimental groups [4]

**Figure 1:**
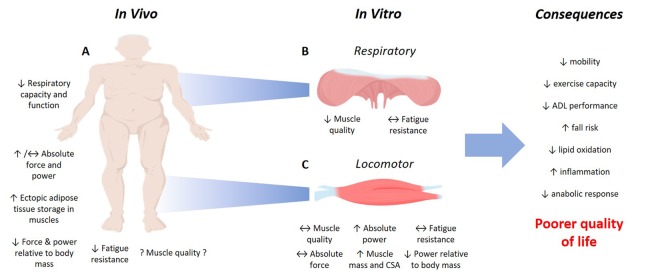
**Impact of obesity in old age on skeletal muscle function.** Excessive adiposity in old age causes a significant reduction in contractile performance in humans (**A**). Less is known about how obesity in old age affects muscle quality (force or power relative to muscle mass), so usage of an isolated muscle model can provide information about the contractile performance of isolated respiratory (**B**) and locomotor (**C**) skeletal muscles, otherwise difficult to examine *in vivo* (observations from Hill et al., 2019). Whilst the studies of *in vivo* and *in vitro* muscle performance do not reciprocate one another, the overall consequence of the altered contractile performance of old obese skeletal muscles is a poorer quality of life. ADL, Activities of daily living. Images from BioRender.

Recent research from our lab [7] examining old (79-week-old) dietary-induced (9 weeks high-fat diet) obese mouse soleus, EDL and diaphragm contractile performance in relation to age-matched control muscles has indicated that absolute force of soleus and EDL was not different between the experimental groups, but absolute power was elevated following a high-fat diet for these muscles. Conversely to that shown using a young animal obesity model [4], muscle quality of the locomotor muscles was unaffected by obesity, however, elevated fatness did exacerbate the age-related reduction in diaphragm muscle quality, which is likely to have implications for respiratory function. In sum, these results provide initial evidence inferring that obesity may accelerate the age-related decline in the intrinsic force producing capacity of respiratory, but not locomotory, muscles (Figure 1)..

Future work is required to understand the effects of chronic obesity (greater than the 9 weeks used in our study on isolated muscle function to provide a more accurate representation of long duration age-related weight gain. This, in combination with work examining the mechanistic changes that account for obesity-associated changes in aged muscle function, is important in developing therapeutic strategies to combat the detrimental effects of sarcopenic obesity on health-related fitness.
